# UK vs US physician decision‐making in the treatment of haemophilia

**DOI:** 10.1111/hae.13766

**Published:** 2019-05-05

**Authors:** Christopher C. Lamb, Adrian Wolfberg, Kalle Lyytinen

**Affiliations:** ^1^ Weatherhead School of Management Case Western Reserve University Cleveland Ohio

**Keywords:** decision‐making, haemophilia, physician decision process, US HCS vs UK HCS

## Abstract

**Introduction:**

Patient–physician shared decision‐making (SDM) has become increasingly seen as having a positive effect on management of chronic diseases. However, little is known of the factors that encourage SDM or how effective it may be at improving health outcomes or how cost‐effective it is.

**Aim:**

To investigate the uses and applications of patient physician–SDM in the management of haemophilia and the influence of healthcare systems in the United States and the United Kingdom.

**Methods:**

This was a qualitative study based on interviews with treatment experts in the United States and United Kingdom. A grounded theory approach was used to analyse the data from the transcribed interviews and themes that emerged as related to the decision influencers. Twelve physicians from each country were interviewed by the author.

**Results:**

Treatment guidelines were viewed as having only limited applicability because of the lack of universal best options in haemophilia. The US physicians in the sample appeared to be more influenced by patient preferences than physicians in the UK, who instead tended to follow policies and standards of care more closely. Physicians in both countries commented that many of their patents had become highly knowledgeable of their bleeding disorder. US physicians were sometimes limited by insurance company policies but also reported that they were often successful in appealing insurance decisions.

**Conclusion:**

The research suggests that there are different influences on decision‐making between healthcare systems; patients and overarching healthcare systems play a major role in how physicians treat haemophilia.

## 1 INTRODUCTION

Recently published haemophilia decision‐making models of care promote ‘shared’, ‘deliberative’ or ‘tailored’ techniques to optimize treatment.^1^, ^2^, ^3^, ^4^, ^5^, ^6^ These models reflect a shift from a paternalistic model of medicine to a process whereby healthcare providers and their patients make treatment decisions jointly. This process of shared decision‐making (SDM) has been defined as an ‘approach where clinicians and patients share the best available evidence when faced with the task of making decisions, and where patients are supported to consider options, to achieve informed preferences’.^7^ Haemophilia is a good example of a lifelong chronic disease that requires physicians to make highly skilled decisions and prescribe costly treatments. The costs to healthcare systems of treating haemophilia can vary greatly between countries as is the case with many high‐cost diseases.^8^ Physician decision‐making may best be considered in the contexts of the specific disease treated and the local healthcare system.^9^, ^10^ Comparing decision‐making influences on physicians who treat haemophilia in two developed countries with very different healthcare systems may offer insights into how chronic care can be improved.

The UK maintains a sophisticated national healthcare database to track all patients within a single system. The government allocates the use of drugs in partnership with drug companies. Manufacturers offering the lowest price per unit receive a contract to supply a certain percentage of the country's demand, which is estimated based on a national patient registry.^11^, ^12^ Products selected for the tender are determined in a blind auction in which drugs are considered for their safety and efficacy and, once selected, considered as equivalent and interchangeable.^12^, ^13^ In contrast, the US healthcare system is a fragmented, multiple‐payer structure with many public and private insurance providers.^14^ The treatment options in the United States are chosen by payers and specialty pharmacies based on internally approved formularies.^15^ Price control regulations or powerful large buyers such as national tenders are not present.^16^

In the fields of psychology and economics, Kahneman and Thaler have proposed theories of dual process and nudging in human decision‐making.^17^, ^18^ These have been further developed by Croskerry and Ilgen in the context of clinical decision‐making.^19^, ^20^ Depending on the circumstances, physicians will either make intuitive decisions based on their overall experience (Heuristic or System 1) or will take an analytic and effortful approach by carefully considering all available information (Rational or System 2).^17^, ^21^ Intuitive decision‐making is rapid because physicians make use of standard approaches to treatment based on clinical experience, which has been described as ‘illness scripts’.^22^, ^23^ Analytic decision‐making takes up more time and can involve close consideration of quantitative measurements and the medical literature to reach decisions on the balance of harms and benefits of a particular treatment.^17^, ^18^, ^24^

Many other factors can potentially influence decision‐making style including physician attributes such as knowledge, experience, self‐efficacy, uncertainty, communication style, acceptance of new technology, training received, openness to change as well as physician demographic characteristics. External influencers on physician decision‐making include patients, collaborators, healthcare systems for payment, technologies, access to research, cost considerations and organizational context.

The survey and analysis described in this report aimed to explore the types of decision‐making used by specialist physicians who treat haemophilia. A qualitative grounded theory approach was used because this method aims to construct theories grounded in qualitative data, as opposed to testing existing hypotheses.^25^, ^26^

## 2 METHODS

### 2.1 Participants

Haematologists with experience treating haemophilia in the United States or the UK were eligible to be included. Participants were offered a small payment for their time. Participants, who were all specialists with expertise in haematology and affiliated with comprehensive treatment centres for caring with patients with haemophilia, were identified by personal acquaintance and networking.

### 2.2 Data collection

Data were obtained through telephone interviews (N = 24) between May and September of 2015. The average length of an interview was 60 minutes, and all were recorded and transcribed verbatim by a professional service. Interviews were semi‐structured in that an interview guide (shown in the Appendix S1) was used but the interviewer could probe topics of interest as these emerged.

Reliability and validity of the interview design and interpretation of findings were addressed based on standard guidelines for qualitative research.^27^, ^28^ The interview protocol was pilot tested on the first participant, and the questions used were assessed for clarity, appropriateness and relevance. Revised interview protocols were created after the initial feedback. Direct quotes from participants were used to support findings.

### 2.3 Data analysis

Grounded theory is a research methodology that aims to construct theories grounded in data, ‘rather than deducing testable hypotheses from existing theories’.^29^ Data collection and analysis occur simultaneously to best identify themes, and guide data collection throughout the process.^30^ Data analysis in this study began immediately after the first (pilot) interview and continued throughout the study. Categories were added and modified as new meanings emerged from the data. A diagram illustrating how grounded theory coding was performed in this study is shown in the Appendix S1. The coding process started with line‐by‐line indexing of the responses using the NVivo version 11 software package.^31^ Similar and equivalent codes were then combined manually. Then, conceptual themes were generated, based on a categorical analysis of the most common codes. A negative case analysis^32^ was conducted to detect any potentially contradictory findings. At the final stage (selective coding), one theme was chosen as the primary category to drive the story of the data.

### 2.4 Ethical assurances

Prior to any data collection, the project was reviewed and approved by the Case Western Reserve University Weatherhead School of Management and an institutional review board (IRB) for compliance with ethical standards, which included US data protection and privacy rules. Informed consent was obtained at the start of each interview: participants were informed about the purpose of the research, the means of ensuring anonymity, and that the survey data would be used in the current study only. Survey data, which contained participant identifying information, were kept in a locked container only accessed by the researcher. Participants were assured that they had the option of withdrawing from the study at any point.

## 3 RESULTS

### 3.1 Enrolled participants

Fifty‐two invitations were sent to haematologists via telephone, email and postal mail. Twenty‐four accepted, one declined, and 27 did not respond. All those who accepted (12 UK based and 12 US based) were interviewed by the first author (CCL). Participant characteristics are shown in Table 1. All interviewed participants described treatment criteria, approaches to patient treatment and the organizational context of their decision‐making. The themes that emerged from the codes are summarized in Table 2. The themes can be grouped into two categories: external factors that affect decisions; and relational factors that are involved in decisions. Of these, the intuitive decision‐making and nudging category were determined to be primary, thereby suggesting that dual process and nudging theories are applicable concepts to understand physician decision‐making across the two different healthcare systems. No contradictory findings could be detected in the analysis. Enrolment was stopped at 24 participants when it was evident that data saturation had been reached and no further themes were emerging.

**Table 1:** Participant details

Category	United States	United Kingdom	Total	% (of 24)
Total	12	12	24	100.00%
Male	9	10	19	79.17%
Female	3	2	5	20.83%
Experience, y (mean)	28	23		
Experience, y				
7‐14	2	1	3	12.50%
15‐24	6	3	9	37.50%
≥25	4	8	12	50.00%
Patient age group				
Paediatric	0	3	3	12.50%
Mostly paediatric	2	4	6	25.00%
Mix	7	2	9	37.50%
Mostly adult	2	0	2	8.33%
Adult	1	3	4	16.67%
Haemophilia treatment centre				
Yes	12	12	24	100.0%
No	0	0	0	0.00%

**Table 2:** Extracted themes

Categories	Descriptions
Intuitive decision‐making	Decisions driven by experiences treating patients including treatment protocols and prescribing in their training, practice, and clinical trials
Limitations of evidence‐based medicine	Scepticism towards decision‐making that strictly follows a standardized procedures and protocols assigned by the organization (hospital, clinic, government, professional body) or published in the literature
Patient‐centric approach	Decisions influenced by the patient such as input, treatment preferences, lifestyle, and response to care
Power difference (Social identity)	Decisions susceptible to the power differences between parties (status, information, etc)
Nudging	Physicians guiding decisions meant to encourage specific routes
Organizational policies	The extent physicians follow policies created by their government and healthcare organizations

### 3.2 External factors

#### 3.2.1 Theme → The philosophical approach to patient‐centric care differs between US and UK physicians

The interview results suggest the US and UK physicians see the role of patients differently when setting treatment goals. The US physicians were patient driven and were influenced by the patient input more than the UK physicians were (Table 3). Participant US#01 would support patients staying on a particular product if they wanted to and if the product was an acceptable alternative. In other words, the US physicians applied SDM in the treatment of haemophilia. On the other hand, UK physicians encouraged the best method of treating haemophilia via prophylaxis as established in guidelines. Participant UK#06 described how patients were usually willing for the physician to make the decision on when to switch from on‐demand treatment to prophylaxis.

**Table 3:** Themes and quotes

Category	Theme	Quote
External factors	Approach to patient care differs between US and UK physicians	[Response to questions regarding decision‐making with changing treatments and technologies] It's been pretty consistent the way we've approached it. There have been a number of times when I felt like there was more than one option that was acceptable to me, so that would have been one. There for a year or two I thought it was reasonable for patients to stick with cryo if that's really what they wanted to do. Then there have also been times when I felt like there was a clear advantage to the new technology. An example of that would be when the recombinant Factor IX product, BeneFIX, became available. I felt we had a long enough track record with recombinant Factor VIII, and we knew that it was 100 percent safe, and we knew that it was efficacious, and just based on the efficacy trials of BeneFIX, that it worked very well, then we pretty much switched all of our Factor IX patients over to BeneFIX. That wasn't something where I said, "Well, this is something you can consider. Here's what you're going to do." –(US#01)
	Approach to patient care differs between the United States and UK physicians	[in response to a question about on‐demand vs prophylaxis] …There'd been no barrier to that so patients that wanted prophylaxis, they could receive that so if I was seeing a patient as a registrar at that point and they were having bleeds, I would suggest to them for the breakthrough bleeds that they go to prophylaxis. They were more than happy that I made that decision and switched them over to prophylaxis from on‐demand treatment. …It's pretty much always been … said to the patient that they should go on to prophylaxis rather than the patient asking for it. Patients are generally who are not on prophylaxis have been reluctant to take regular back to concentrate. However, we've always supported that and suggested that's the best treatment in all the time I've been working in haemophilia. We tend to suggest then with your breakthrough bleeds, you're going to get a target joint. The best thing for you to do is to switch over to prophylaxis and take it more frequently. It's an ongoing battle. We're doing it all the time. Then they stop the prophylaxis and then telling them to restart prophylaxis. Tends to be a common situation rather than a one‐off conversation. –(UK#06)
	EBM standards are flawed or missing	[In response to a question about decisions when the participant was younger] Some of them was during my fellowship so certainly I had faculty help in decision‐making. We had a very large haemophilia population and so I spent additional time in the haemophilia clinic during that time since I knew that was what my interest was. I guess… [In response to a question about challenges and decision‐making using guidelines/protocols] I think haemophilia is one of those areas that there's not a lot of evidence‐based guidelines so it's based on experiences, yourself and those around you and so I think I've learned most of my, I got it through [Dr *] and I know he treats patients in a certain way and that's how I learned, to treat them just by following his example…‐(US#10)
	EBM standards are flawed or missing	[In response to a question that categorizes physicians as experience or data‐driven] In between those 2 options? I'm fully aware of the data that is published. I get the main specialty journal anyway called haemophilia and so I know what the evidence is, I go to enough meetings to hear what is the evidence, but I do also, I'm also fully aware of the limitations of the evidence because most of the evidence base in haemophilia is poor. People do not appreciate how poor the evidence based on haemophilia is. They believe that if you see a clinical trial that you believe what it says, but actually it's a very selective group of patients with a selective indication. –(UK#11)
	Organizational policies	[when asked to describe the treatment process] I think in the UK, we make a decision on treatment [across] the board, across the country, we have a national organization in the UKHCDO, so treatment policy is decided by that body. We decided a long time ago that recombinant factoring should be the treatment of choice for all of our patients. We're now in a situation where we only use recombinant FactorVIII and FactorIX for our patients with haemophilia. This was a decision which was driven, obviously, by HIV and HCV. Perhaps, particularly in the UK, also because of issues related to variant CJD. … Certainly for the last decade, all the patients here in the UK have been treated with recombinant. As regards to products, we have a national tender. The national tender effectively decides price and volume of each product that's used in the UK. I still have the right to use whichever product I want, but in reality, I negotiate with my colleagues. It would be stupid to use large quantities of anything other than the cheapest products. –(UK#01)
	Organizational policies	[when asked about coverage of certain drugs] Most hospitals there's always somebody who's in charge of these super expensive drugs, it's usually like a pharmacy person plus a doctor of some sort. But I've never been lucky in the current situation that I had to authorize Novo Seven, but if I wanted it on someone and someone else is going to authorize it, and you needed to get it, you would try to push the doctor and the pharmacists and try to impress upon them the clinical need, and get it that way. ‐UK#10
	Organizational policies	[when asked about insurance coverage and restraints] Not so much, because the insurance carriers usually know that this person is haemophiliac and there's haemophilia centres especially with the patient as you know. But in, more in a situation, so where you have to use Novo Seven for example, that is a problem in America too because the hospital ends up eating the cost and there is never time to kind of get pre‐authorized to use that because you're using it in a true emergency and you're… basically the hospital eats the costs if you don't get reimbursed. So, there's a lot of pressure not to use Novo Seven, even here. [when asked if there is a workaround] Most hospitals there's always somebody who's in charge of these super expensive drugs, it's usually like a pharmacy person plus a doctor of some sort. But I've never been lucky in the current situation that I had to authorize Novo Seven, but if I wanted it on someone and someone else is going to authorize it, and you needed to get it, you would try to push the doctor and the pharmacists and try to impress upon them the clinical need, and get it that way. ‐UK#10
	Organizational policies	[When asked if coverage was an issue] The issue they came up with us was a patient who was at that time on a state program who had switched then to an adult program and he was put into a managed care system and he was put into a managed care system and a managed care system had an idea of what they wanted to do which was not what I wanted to do. I complained to the state and first the managed care system threw the towel and then the patient was reassigned to of the managed care plan, that option was available, now the patient is on private insurance. [when asked if coverage was ultimately provided] Yes. ‐ US#02
	Organizational policies	…we diagnosed a case of PNH, paroxysmal nocturnal haemoglobinuria, and we've gone to the county to get them, based upon all the literature and all the benefit, to pay for eculizumab. Okay? $400,000 a year for the patient. Okay? Because in fact the data says that the 5‐year mortality is 30%, the 5‐year mortality of the patients on the drug is less than 5%. Okay? How can you refuse it? [when asked if the participant win appeals and how often they occur] Oh yeah… I suspect that for the whole division we probably do appeals about 4 or 5 times a year for various indications… They don't want to put it on the formulary because they don't want to make it a routine available, nor do they want to make it a restricted formulary because they're afraid that even the restricted pattern… you go to the appeal and you usually can get the drug. –(US#04)
	Organizational policies	[when asked about being cost conscious] I suppose subconsciously it probably does. I don't sit there and try to wonder what's going to be paid for and what isn't. On the other hand, if I think one approach is going to cost 75% more than another, I wonder whether I'm going to get push‐back from the insurers and have to write a whole bunch of letters and spend hours of time trying to get approval. … I already do have approved treatments. I would say that in the back of my mind if I think that part of me wants to give a treatment, as long as I think it's effective at the lowest possible price just from my own consciousness, but I suppose that is influenced to some extent to not wanting to get hassled by insurance companies and have to spend extra time justifying something. US#06
Internal factors	Intuitive decision‐making	[Interviewer asked about mentors and clinical experience] I think early on it was from mentors because… I was rather inexperienced. At that stage, I had the necessary specialist qualification, but still lacked experience. Experience counts for an awful lot. I was very much influenced by mentors and senior colleagues, probably more than clinical trials… Then I think number two importance comes clinical trials because [clinical trials] are rigorous, logical and have, one hopes, believable outcomes…Number three comes the trial and error. –(UK#07)
	Intuitive decision‐making	[In response to a question about adopting long‐acting products compared to recombinant in the past] Maybe, maybe in the beginning: As more information comes out, my being somewhat tentative about its [long‐acting products] use doesn't seem to be warranted by the facts. I would say my feeling now is, this stuff is great. You can, if you can use, if you can stick the veins less often, that's a good thing. Now I don't think you get that much because of the nature, the Factor‐8 protein. If you use a long‐acting 9, then you really have reduced the frequency of infusions to patients on a prophylactic schedule than Factor‐8. Is that important in a kid where 1 less stick per week is important, yes; but maybe, less important in an adult. ‐(US#05)
	Power balance between patients and physicians is mostly influenced by health literacy	[when asked about switching a patient to extended half‐life products] … what I say is that "I need to know what your pharmacokinetics are on your current product because if you are on the top side of the kinetic curve using the current product that you're on, and if you have an active lifestyle so that you are going to be active 3 out of 5 or 7 days out of the week, then I need to know how to keep your clotting factor level over 15% at least so that you can be physically active and I need to know how long that area under the curve is going to be." ‐#US03
	Power balance between patients and physicians is mostly influenced by health literacy	[Response to questions regarding influence from patients] I started to become I think more aware that probably around … '85 through '90. They had a big haemophilia program over at [Hospital], which is now part of Penn but then wasn't. A lot of the patients went there. We did rotate a little bit over there. I would say most of that population was not that really educated as sort of an inner‐city kind of group. I think we were trying to just use newer products that had the potential to be safer, even though as I recall we were never really quite sure. Same was true when I was at [other hospital] for 5 years. ‐(US#06)
	Power balance between patients and physicians is mostly influenced by health literacy	[when asked if a patient has come in and requested something and you disagreed] I hope that my relationship is good enough with these families that if they come in… Rarely do they demand to be switched. They may request that they discuss it. They may say, "Can we talk about this product X." We'll sit down, we'll discuss it. Now if they have a very strong opinion about it and I feel that it's well placed and that they have a good information base on that and they understand risk and the benefits, then I hope that I'm open minded enough to go ahead and work with them in addressing that. If we both agree, then I'll go ahead and write the script. I just want them to make informed decisions. I don't want them to either see an ad, go to a meeting and be approached by a sales person or something like that and come back and say, "Oh, I need to switch because so and so told me to switch." ‐US#08
	Power balance between patients and physicians is mostly influenced by health literacy	[Response to questions about patient involvement in decisions] It was a joint decision. In other words, as I'm sure you know, patients with haemophilia in general, not all, are very complicated and knowledgeable to their disease. They know more than us, for instance they can see the doctor. "I can tell you that I am bleeding." I learn too they were right, and I was wrong. There was no sign in any way. I learned to very soon to take their advice and their opinion. Of course, it depends on the culture of the people because in general they are very cultivated. They take part in the decision‐making. I think the switch came prior to consider it was obvious that the reason that I told you because it involved them to come to the hospital. Now the home treatment, it was easier, quicker for them. Even when they came to the hospital, it was a matter of infusing them instead of an hour or so to prepare cryoprecipitate. –(UK#03)
	Power balance between patients and physicians is mostly influenced by health literacy	[when asked about disagreements with patients] …One or two of them have simply preferred what they know, and they've remained on plasma and remained relatively well. A lot of them do have poor quality of life because they've lived with the disease for decades and are struggling with the consequences of that. To me, that's never a dispute. That's just a patient saying, "Yup, I'll stick with what I know." –(UK#07)
	Physicians nudge decisions	[Response to questions about patient choices] Initially they have to trust you but as they become very experienced as patients, they often do suggest treatments and so on and that's what can be very difficult when you say "Now, I don't think that's the right treatment for you", is what you don't want them to have the sort of relationship where they will claim "You're just saying that because of cost." If you have a patient that… sometimes you have to change them from one factor to another and if you have one that says, "I'm not going to change because I don't think that the other one works", you just keep them on… If there was no medical reason to change, you would then keep them on the other product, so I think there would be a… they would have a choice. I was always very lucky with the children in that they were the first patients to go on to recombinant because they hadn't been exposed before, so that was worked well. –(UK#12)
	Physicians nudge decisions	Part of the annual visit is to vote into new products. Sometimes the patients will bring up the question, "Doc don't you think I'd be a good candidate for these products?" My own philosophy has been the following. … "I need to know what your pharmacokinetics are on your current product because if you are on the top side of the kinetic curve using the current product that you're on, and if you have an active lifestyle so that you are going to be active 3 out of 5 or 7 days out of the week, then I need to know how to keep your clotting factor level over 15% at least so that you can be physically active and I need to know how long that area under the curve is going to be." Because the prolonged half‐life products most patients don't appreciate only talks about the prolonged area under the curve. It does not talk about how long the peak activity is sustained. So when you begin to talk to patients and you get them to understand that the benefits of the extended half‐life products are only at how long will you remain over 1% of Factor‐8 clotting factor activity, they begin to understand that maybe they're not the ideal candidate to be on these products because what they really need is to have over 30% activity so they can go out to soccer practice 3 times a week and not have a bleed. –(US#03)
	Physicians nudge decisions	If it's a brand‐new patient, usually there are parents involved as well. Generally speaking for haemophilia A, we would just go for recombinant product and we'd give them information for recombinant product and actually we wouldn't give them any information on plasma‐derived.In that space, specifically spoke about plasma‐derived, wouldn't mention it. –(UK#09)
	Physicians nudge decisions	[when asked about patient choice of product] Very little. The patient really, the more patients know what they're product is, sometimes the parents know because a lot of the patients are children, so they know what their product is, but that's about it. They don't have too much choice, and that, again even here, the patient can tell you what product they don't like, what product they like, but it's ultimately what they get is basically dependent upon what the centre, the treatment doctor and the centre decide. –(UK#10)

#### 3.2.2 Theme → Evidence‐based medicine standards are of limited applicability or lacking altogether

Although decisions are reinforced by data and literature, the evidence‐based medicine standards for the treatment of haemophilia were considered lacking (Table 3). Participant US#10 stated that there although evidence‐based medicine (EBM)‐based guidelines are available, these alone are insufficient for decision‐making, and one must rely on experience and training for the nuances of care. Participant UK#11 noted that EBM in haemophilia is of limited applicability in clinical practice because published clinical trials are very contextual and the patients in the trials are not necessarily representative of the patients they treat in practice. EBM was considered not well matched to patients’ individual needs. For example, participants were eagerly anticipating the introduction of next‐generation extended half‐life factor IX products although these are likely to be mainly relevant for young active patients. Currently available extended half‐life products were considered sufficient for more sedentary patients who are less at risk from lower trough levels of clotting factor.

#### 3.2.3 Theme → Organizational policies

In the UK, physicians appeared to follow policies more closely than US physicians. Additionally, UK policies are created and enforced by a tender board, which may account for their more standardized care compared to the United States; tender board members include haematologists and patient representatives and are selected by the Department of Health, the Commercial Medicines Unit, and the United Kingdom Haemophilia Centre Doctors Organisation (UKHCDO).^12^ Participant UK#01 stated how this affects the standard of care for use of recombinant factor VIII and factor IX.

On the other hand, US physicians were sometimes restricted by insurance company policies but also reported that they were often successful in appealing insurance decisions (Table 3). For example, participant US#04 described a case where the covered treatment for paroxysmal nocturnal haemoglobinuria (PNH) had a 30% associated mortality rate in 5 years, whereas the treatment US#04 sought had a 5% associated mortality rate; the difference was the cost of the treatments. The request to use the safer, more expensive treatment was rejected by the insurance company. The physician then successfully appealed this denial. Likewise, participant US#06 stated that they would consider the marginal benefits that might be gained by switching to more effective treatments; US#06 had to determine whether the effort of going through an approval/appeal process was justified by the benefits of a treatment that had been denied by an insurance company. For US participants, the effort needed for a successful appeal seemed to be a potential barrier even when they were confident that they would ultimately win in this situation.

### 3.3 Internal factors

#### 3.3.1 Theme → Intuitive decision‐making

Physicians appeared to rely on intuitive decision‐making for the treatment of haemophilia. They emphasized their experiences in training, education and experience with therapeutic agents and treatment protocols although considered that all their decisions were supported by published data. For instance, Participant UK#07 (Table 3) emphasized the experience in haemophilia gained from mentors. In some instances, such as described by participant US#05, physicians restrict their prescribing of new products until clinical data have accumulated sufficient evidence showing improvement over existing treatments.

#### 3.3.2 Theme → Power balance between patients and physicians is mostly influenced by health literacy

The balance of decision power appeared to be mainly influenced by the asymmetry of information between physician and patient. Participant US#05 emphasized that the physician is necessarily ‘a source of a lot of information that the patient may not have or the families may not have’. When patients ask about new products, participant US#03 described advising patients on the intricacies involved with switching products. However, patient perspectives could often be valuable. Participant US#06 stated that patients in haemophilia treatment programmes at ‘hospital A’ were much more informed and knowledgeable than patients at ‘hospital B’ were, and their input and preferences were consequently given more weight during decision‐making. Participant UK#03 stated that some patients were on occasion able to persuade them that physician decisions were incorrect, especially if they were very knowledgeable about their condition.

#### 3.3.3 Theme → Physicians nudge decisions

Physicians from both countries appeared to nudge patients’ decision‐making for the treatment of haemophilia. Participant UK#11 tended to limit information given to patients if it were not among the available treatment options. Participant US#03 questioned the improvements that new products can offer, addressing the limited scientific basis behind the new extended half‐life agents (otherwise described as long‐acting). Participant UK#09 explained their approach with new patients, specifying that they generally avoid mentioning plasma‐derived products to them. Participant US#05 specified that patients needed to demonstrate how well they understood the product options available to them.

## 4 DISCUSSION

This study provides insight into the decision processes of US and UK physicians who treat haemophilia. Table 4 provides a summary of similarities and contrasts between US and UK physicians in decision‐making.

**Table 4:** Summarized comparison between US and UK physicians

	United States	United Kingdom	Similar?
Patient‐centric approach	Patient‐driven	Disease‐driven (standards from system)	No
Flaws of evidence‐based medicine	EBM useful, but contextual	EBM useful, but contextual	Yes
Organizational policies	Follows or adjusts treatment based on reimbursement (insurance)	Closely follows NHS developed standards for care	No
Decision‐making style	Intuitive over heuristic	Intuitive over heuristic	Yes
Power difference (social identity)	Patient influence depends on patient knowledge	Patient influence depends on patient knowledge	Yes
Nudging	Question and discourage changes for only marginal improvements (persuasion)	Limit information was given to the patient if it is not in their available options for treatment (omission)	Yes

Abbreviations: EBM, Evidence‐based medicine; NHS, National Health Service.

Physicians in the US appeared more likely to make decisions based on patients’ needs, whereas the UK physicians tended to be more influenced by colleagues and government policies. This ‘mutual participation’ occurring in the United States is the essence of SDM.^33^, ^34^ The US participants would work closely with a patient to inform them of suggested treatment options while also coordinating with their insurance company. In some instances, this involved the physician personally contacting the insurance company: in their experience, this was successful in every case (Table 3).

On the other hand, the UK approach to patient care was found to be more ‘disease‐oriented’^35^—patients are treated according to standard disease guidelines adopted by organizational policies, which could be refined according to context. These treatment patterns parallel an overarching tendering system in which all patients access to a uniform choice of recombinant agents, except for a minority who receive plasma‐based agents. UK physicians generally concurred with current treatment standards and rarely considered other options such as plasma‐derived agents. The restrictions encountered in the US were, in some respects, the equivalent of the UK product tender policies; when a UK physician seeks alternative treatment options for a patient, they must get special approval or risk the hospital incurring the cost of the care (see UK#10 in Table 3).

Evidence‐based medicine standards for the treatment of haemophilia were considered insufficient for effective patient care. Participants in both countries understood the importance of being up to date with EBM standard literature, but often warned about the reliance on study data alone because the context of those studies was often not relevant to their patients. Participants described an intuitive as opposed to EBM approach to treating patients, especially for initial treatment and changes to treatment. Both groups also reported staying up to date on the literature to ensure the patients were getting the optimal treatment according to their specific needs.

Responses revealed that there is a dynamic balance of decision‐making power between patients and physicians in both countries. The physicians may have the status and knowledge to dominate the initial diagnosis and treatment decision but patient decision‐making becomes more relevant, especially in regard to adjustments and corrections to treatment after recurring visits once the patients have closed the knowledge gap and are more aware of their treatment needs. Experienced patients will also initiate discussions on treatments. Physicians in both countries were experienced when it came to nudging patient decisions. In the United States, nudging was more likely to be achieved by providing ample information on the physician's preferred choice. In contrast, in the UK, physicians may omit information on options that are non‐standard or not included in the tendering system. In the UK, the nudging process is reflected in the healthcare system: the master list of products to be available for tender is determined by the votes of the UKHCDO selection panel and haematologists throughout the UK, thereby placing the physician at the beginning and end of a decision nudging process for available treatments, although a patient representative does sit on the selection panel.

### 4.1 Implications

Shared decision‐making is considered the ‘pinnacle’ of patient care.^36^ However, its applicability in the management of specific chronic diseases such as haemophilia and its potential for improving the quality of care is not well studied.^37^, ^38^, ^39^ Concepts from Kahneman and Thaler may be useful to better understand the factors that influence clinical decision‐making, and these could be used to further improve haemophilia decision‐making models.^1^, ^2^, ^3^, ^4^, ^5^, ^6^ The results of the study may also be of interest from a policy perspective in understanding the influences and barriers that physicians experience when making treatment decisions for the management of haemophilia and may be applicable to other disease states with high treatment costs. Findings may also be a relevant in physician self‐assessment of decision‐making styles and biases and to offer insights into how chronic care can be improved.^40^

Limitations of this study include those inherent in the sampling which might not be representative of American and British physicians who treat haemophilia. The grounded theory approach meant that sampling was continually redefined as data were collected and analysed. Despite this, the physician sample consisted mainly of Caucasian men. As with any study using phone interviews, it was limited to physicians with time available on their schedule. The open‐ended nature of the interviews, while encouraging discussion of themes of interest may also have excluded some physician experiences altogether. It is possible a survey including more physicians and a more diverse group would have dissimilar findings to this one. Further insights on the decision‐making and nudging processes might also be gained through a retrospective analysis. Empirical findings obtained in this way could be integrated into existing decision‐making models^1^, ^4^ with the aim of improving medical consultations or ‘conversations’ in haemophilia care or other chronic conditions. Likewise, the limitations of EBM could be evaluated in the delivery of precision medicine and outcomes‐based care.

In conclusion, this interview‐based study found that decision‐making in American and British physicians who treat patients with haemophilia was guided by experience (intuition and nudging) and training. EBM standards were shown to be widely recognized in both countries although considered to have limited applicability to real‐life clinical decision‐making. Patient healthcare literacy was generally thought to enhance patient participation and overall decision‐making. The comparisons between two countries showed that healthcare system itself had a considerable influence on physicians’ decision‐making.

## DISCLOSURES

The Dr Lamb has provided consulting services to biopharmaceutical companies that develop, manufacture or market haemostasis products which could be used in the treatment of haemophilia. These companies include Talecris, Omrix, ProFibrix, Sealantium, Omri, GC Pharma and Kedrion, SpA. Dr Wolfberg and Dr Lyytinen have no interests that might be perceived as posing a conflict or bias.
