# Supplementary material for: UK vs US physician decision‐making in the treatment of haemophilia
Source: Haemophilia. 2019 May 5;25(4):616–25. doi: 10.1111/hae.13766 (PMC6850192; doi:10.1111/hae.13766)

# Appendix

Interview Protocol and Questions

1. Introduction and Explanation—read to the interviewee before proceeding
   1. Greeting 🡪 “Hello [*name of physician*]. Thank you for taking the time to meet with me today. Your participation is greatly appreciated. Before getting started, there are a couple things I would like to cover.”
   2. Purpose and Format of Interview 🡪 “As a current student in the Case Western Reserve University Doctorate of Management (DM) program, I am interested in developing a greater understanding of the physician decision process regarding haemophilia. I will ask you a series of open-ended questions on this topic, and I will ask one or more follow-up questions as you respond. The interview will last approximately 60 to 90 minutes.”
   3. Confidentiality 🡪 “Everything you share in this interview will be kept in strictest confidence, and your comments will be transcribed anonymously—omitting your name, anyone else you refer to in this interview, as well as the name of your current organization and/or past organizations. Your interview responses will be included with all the other interviews I conduct.”
   4. Recording 🡪“To help me capture your responses accurately and without being overly distracting by taking notes, I would like to record our conversation with your permission. Again, your responses will be kept confidential. If at any time you are uncomfortable with this interview, please let me know and I will turn the recorder off.”
   5. “Do you have any questions before we begin?”
2. Interview questions
   1. Please introduce yourself.
      1. Name
      2. Current job title and responsibilities
      3. Education/training
      4. Years of experience
   2. Please summarize your experience with haemophilia in your practice
      1. How often do you treat haemophilia?
      2. How long have you treated such patients?
   3. Walk through how you currently decide a treatment for haemophilia.
      1. Probe for how this process was initially formed.
      2. Probe for specific sources of information that contributed to this process.
      3. Describe factors that impact this process.
      4. Compare this process to how you would approach haemophilia when you first started.
   4. Describe your experience with new technology
      1. Tell me about the change
      2. What was your personal attitude towards the new technology and the changes it may have brought?
      3. What were the influences for or against the change?
      4. What social norms towards change and new technology?
   5. Describe any factors that may facilitate or prevent the implementation of treatment.
      1. Probe for internal factors
      2. Probe for external factors
   6. Describe how other physicians decide on a treatment of haemophilia.
      1. Probe for the influence of peers has on their decision process.
   7. What is the role of the patient in treatment?
   8. Are you involved in clinical trials?
   9. Do you trust the data or experience more?

Appendix Figure:

Schematic example showing coding process involved in the qualitative analysis of quoted statements from immunologists


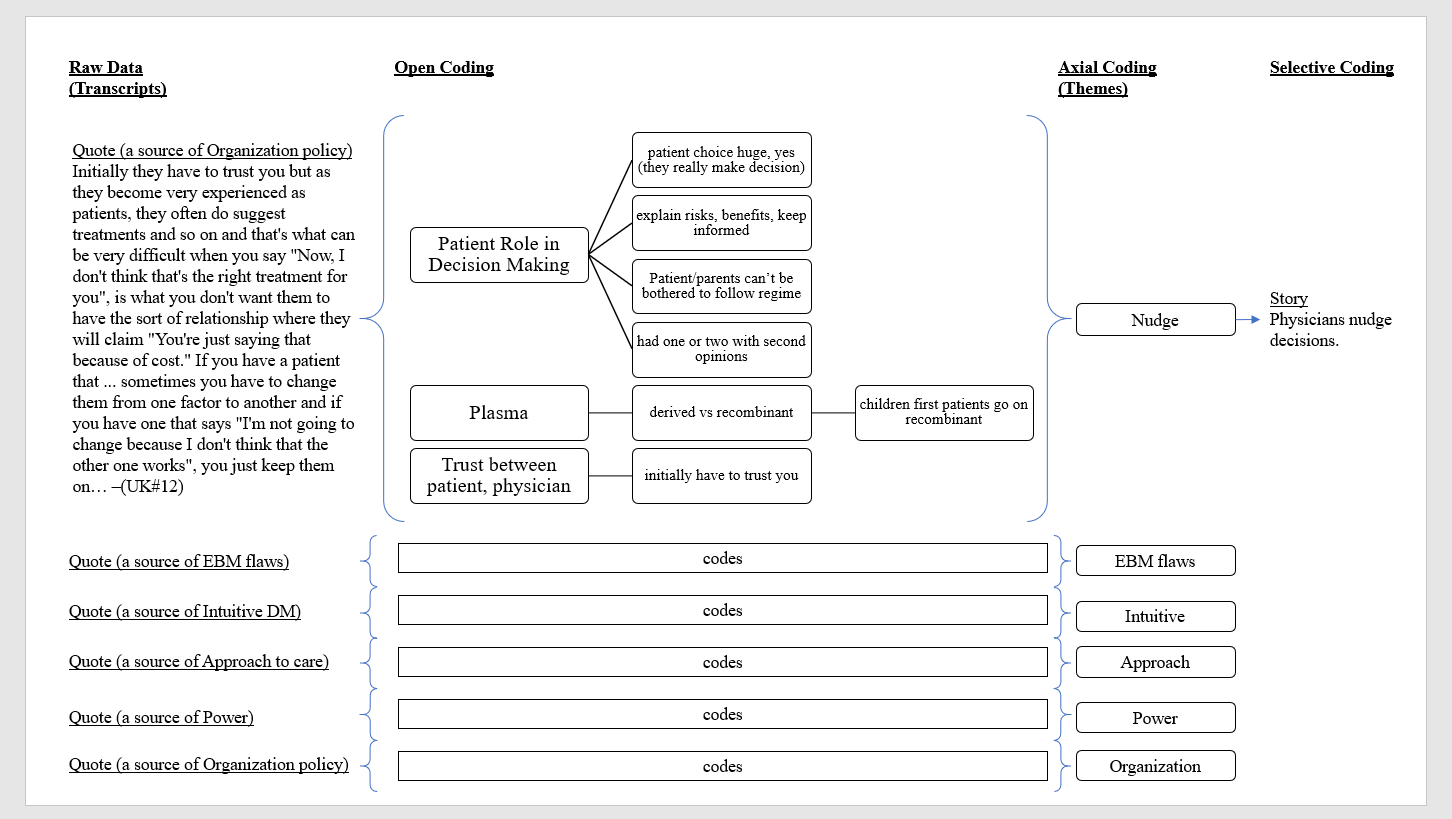

Supplement: Supplementary file 1 [file HAE-25-616-s001.docx]
